# A reflection on enzyme-coupled supramolecular sensing: overcoming selectivity barriers with macrocyclic reporter pairs

**DOI:** 10.1039/d5sc90224j

**Published:** 2025-10-20

**Authors:** Shuangqi Song, Yu Liu

**Affiliations:** a College of Chemistry, State Key Laboratory of Elemento-Organic Chemistry, Nankai University Tianjin 300071 P. R. China yuliu@nankai.edu.cn

## Abstract

Macrocycle-based supramolecular sensing systems have emerged as a powerful approach for biomolecular detection, yet practical applications remain challenging in inadequate selectivity toward structurally similar analytes, low signal-to-noise ratios, and environmental interference. Liu *et al.* (D.-S. Guo *et al.*, *Chem. Sci.*, 2011, **2**, 1722–1734, https://doi.org/10.1039/C1SC00231G) developed a novel “supramolecular tandem assay” by integrating the *p*-sulfonatocalix[*n*]arene·lucigenin (LCG) host–guest reporter pairs with enzymatic transformations, achieving highly selective, label-free detection of choline and acetylcholine at physiologically relevant micromolar concentrations. The enzymatic step confers molecular specificity absent in conventional macrocycle-based sensors, while the host–dye displacement mechanism provides a tunable signal output. This strategy overcomes inherent limitations of conventional macrocyclic hosts while preserving their broad applicability, providing transformative opportunities for real-time enzyme activity monitoring and Alzheimer's drug screening.

## Abstract 2

In celebration of our 15th anniversary and some of our most popular articles, Yu Liu reflects on the area of supramolecular tandem assays, building on a *Chemical Science* manuscript published in 2011 on this topic [Guo *et al.* (*Chem. Sci.*, 2011, https://doi.org/10.1039/C1SC00231G)].

## Graphical abstract

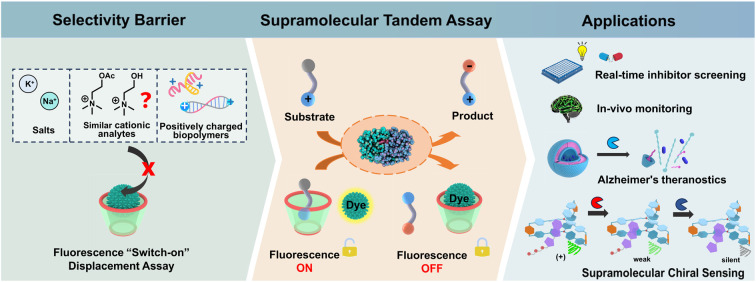

Macrocycle-based supramolecular sensing achieves target detection through highly selective and sensitive molecular recognition enabled by host–guest interactions, with broad applications in biological,^1–3^ medical,^4,5^ and environmental monitoring fields.^6,7^ This recognition capability stems from diverse non-covalent interactions, including hydrogen bonding,^8,9^ electrostatic interaction,^10,11^ and hydrophobic interaction,^12,13^ that provide precise molecular recognition through matching size, shape, and functional group. Among these, *p*-sulfonatocalix[*n*]arenes with high water solubility and biocompatibility exhibit exceptional binding capacity and high selectivity toward various organic cations, standing out as ideal sensing platforms.^14,15^ However, conventional supramolecular sensors demonstrate critical limitations in complex biological environments, particularly insufficient target specificity, poor interference resistance, and incompatibility with dynamic physiological processes, restricting their utility for real-time monitoring of enzymatic activities and detection of disease-relevant biomarkers.^6,16^ The presence of competing ions, non-specific protein adsorption, and pH variations further complicate their practical implementation in biologically relevant conditions.

In 2011, our group published in *Chemical Science* (https://doi.org/10.1039/C1SC00231G),^17^ achieving a significant advancement by developing the host–guest complex of macrocyclic calixarene and fluorescent dye lucigenin (LCG) coupled with enzymatic reactions, enabling highly selective detection of acetylcholine and choline, which successfully addressed the long-standing selectivity challenge in conventional displacement-based sensing platforms. The calixarene·LCG reporter pairs represented a major advancement, exhibiting a remarkable 140-fold fluorescence quenching upon complexation, which was characterized through comprehensive electrochemical and photophysical studies. First, the host–guest complexes were systematically characterized. Job's plot analysis confirmed a 1 : 1 stoichiometry for the calixarene·LCG complex. Fluorescence titration and isothermal titration calorimetry revealed binding constants (*K*_a_) up to 10^7^ M^−1^ between calixarene and LCG. Further structural elucidation through ^1^H NMR titration and X-ray crystallography demonstrated that the host–guest complex stability stemmed from multiple synergistic noncovalent interactions, including hydrophobic effects, electrostatic attraction, and π–π stacking. The optimal size complementarity between the calixarene cavity and LCG not only explained the high binding affinity but also the fluorescence quenching mechanism, namely the precisely regulated electron transfer process facilitated by the host–guest geometric structure upon complex formation. X-ray crystallographic analysis revealed that one acridine moiety of LCG deeply inserts into the electron-rich calixarene cavity, while the second acridine stacks externally against the macrocyclic rim. This arrangement created optimal orbital overlap for photoinduced electron transfer from the excited LCG to the calixarene, as further confirmed by cyclic voltammetry showing exergonic driving forces exceeding −1.3 eV. The rigidity imposed by complex formation further suppressed non-radiative decay pathways, amplifying the quenching efficiency beyond typical donor–acceptor systems. To evaluate environmental interference, *K*_a_ was determined at varying salt concentrations, revealing that the calixarene·LCG reporter pairs still exhibited poor interference resistance.

It is evident that traditional macrocyclic host–guest replacement detection methods face fundamental selectivity limitations, especially when distinguishing between structurally similar cations like acetylcholine and choline, which differ only by an acetate group. To circumvent this limitation, the authors developed an ingenious enzyme-coupled supramolecular tandem assay strategy (STA) (Fig. 1). By integrating supramolecular sensing with enzymatic cascade reactions, they leveraged the absolute substrate specificity of enzymes to transform previously indistinguishable binding events into distinct fluorescent signals. This transformation occurs through enzymatic conversion-induced charge alterations.

**Fig. 1:**
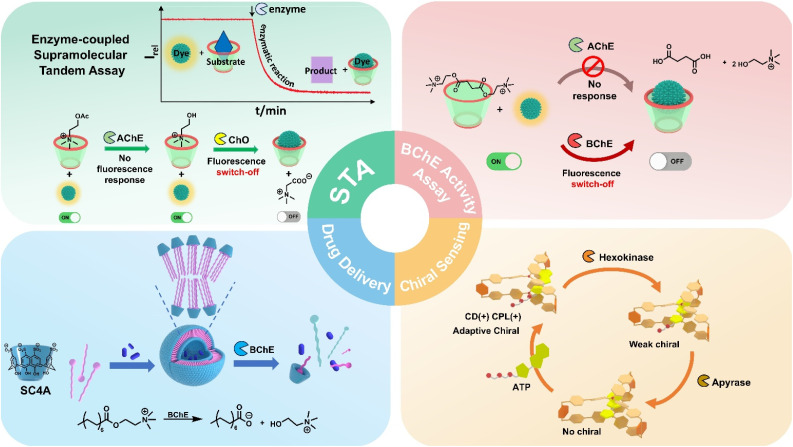
Representative cases of enzyme-responsive supramolecular sensing from our group.^17–19,22^

In practice, the authors first demonstrated the superior performance of the calixarene·LCG reporter pair in amino acid decarboxylase assays, demonstrating performance superior to all previously reported reporter pairs, with higher sensitivity, lower background interference, and greater suitability for high-throughput inhibitor screening. On this basis, they achieved selective detection of neurotransmitter-targeting enzymes, choline oxidase (ChO) and acetylcholinesterase (AChE), while preserving the real-time monitoring capability intrinsic to supramolecular sensing platforms. For ChO detection, enzymatic conversion of the strongly binding choline substrate to weakly interacting betaine triggered LCG release from the calixarene cavity, resulting in a fluorescence decrease that enabled precise determination of enzymatic parameters (*K*_M_ = 160 ± 10 μM). The most innovative contribution was the development of a dual-enzyme cascade system for AChE monitoring, where AChE-generated choline underwent immediate oxidation by ChO to betaine, amplifying the fluorescence response, measuring AChE activity at nanomolar concentrations of both host and dye components, with negligible perturbation of the enzymatic reactions. This supramolecular platform enabled real-time AChE inhibitor screening for Alzheimer's research and absolute quantification of acetylcholine/choline *via* enzyme kinetics (μM detection limits). The innovative STA overcame selectivity limitations of conventional displacement assays by sequentially activating enzymes for stepwise neurotransmitter determination. Merging enzymatic specificity with supramolecular fluorescence sensitivity, the approach provided a label-free, real-time solution for neuroanalysis and drug discovery. Crucially, it operates at nanomolar probe concentrations to minimize interference, enables multi-analyte detection in limited sample volume, and leverages standard plate readers, all while eliminating the need for costly antibody-based methods.

Based on the STA principle, our group achieved specific detection of butyrylcholinesterase (BChE) through substrate engineering innovations and mechanism optimization in subsequent work.^18^ This platform employed *p*-sulfonatocalix[4]arene (SC4A)·LCG reporter pairs and BChE-specific substrate succinylcholine (SuCh). Enzymatic conversion of strongly binding SuCh into weakly competitive choline displaced LCG from the calixarene cavity, inducing a distinct “switch-off” fluorescence response. Notably, the system discriminated BChE from AChE with 7-fold selectivity and remained functional under physiological AChE levels. It enabled real-time BChE activity quantification and nanomolar inhibitor screening, elevating supramolecular sensing from a generic enzyme activity detection platform to a disease biomarker-targeted analytical tool. The core breakthrough is to leverage the substrate specificity of BChE to address the long-standing challenge of cross-reactivity among homologous enzymes. This achievement has established a technological foundation for the supramolecular diagnosis of Alzheimer's disease.

Expanding the scope of this supramolecular strategy, Guo *et al.* developed a host–guest vesicular system based on SC4A and natural myristoylcholine, achieving enzyme-responsive supramolecular targeted drug delivery.^19^ When the vesicles were loaded with tacrine, a therapeutic drug for Alzheimer's disease, the enzymatic cleavage reaction within the BChE-overexpressing lesion microenvironment hydrolyzed myristoylcholine into choline and myristic acid, which disrupted the host–guest interactions, triggering vesicle disintegration and the release of encapsulated drugs. This process not only achieved BChE-triggered targeted delivery but also synchronously monitored drug release efficiency through optical signals, forming a closed-loop theranostic system. Crucially, the released tacrine inhibited BChE activity, automatically slowing subsequent drug release to prevent overdose toxicity. Furthermore, the vesicles maintained stability in physiological saline environments with ultra-low cytotoxicity, establishing a supramolecular theranostic platform for Alzheimer's disease, providing a new paradigm for transforming biomarker detection into precision therapy. Extending the high-affinity recognition of choline derivatives by SC4A, we constructed linear supramolecular polymers *via* α-cyclodextrin (α-CD) threading of a dual-functional axle (suberyl dicholine, DiCh).^20^ This enzyme-responsive disassembly emulated biodegradable polymer behavior while managing degradation products *in situ* through the confinement of α-CD, preventing aggregation, which could be applied to degradable biomaterials, such as scaffolds or tissue scaffolds that require clearance over time.

Label-free supramolecular bioanalysis *via* host–guest interactions is now being extended to chirality-sensitive signals. Cao's group has constructed three chiral supramolecular organic frameworks (SOFs) by cucurbit[8]uril (CB[8]) and tetraphenylethylene derivatives (TPE).^21^ They innovatively proposed a “gear-driven”-type chiral transfer mechanism, where peptides acted as “drive gears”, the linkage units in SOFs as “driven gears”, and TPE units as “output gears”, enabling multi-step chirality transfer from peptides to TPE units. These SOFs can specifically recognize peptides with N-terminal tryptophan (W)/phenylalanine (F) *via* CB[8], exhibit different circular dichroism responses to the same l-type peptides, and can accurately distinguish various peptides by combining with principal component analysis, providing a new system for biomolecular detection related to supramolecular chiral enzyme recognition. In 2024, our group also reported a biofuel-driven chiral supramolecular transfer container based on a hexacationic triphenylamine cage (H) and adenosine triphosphate (ATP).^22^ H can efficiently encapsulate ATP through multiple noncovalent interactions, and ATP can activate chirality transfer, enabling the achiral cage to exhibit circularly polarized luminescence and a positive Cotton effect. Under the tandem catalysis of hexokinase and apyrase, it can stepwise regulate chirality transfer, and its chirality can be recovered upon refueling with ATP, realizing programmable regulation of multistate chiral luminescent supramolecules, which has been successfully applied in chiral logic gates and multilevel information encryption, providing new insights into intelligent supramolecular sensing.

## Conclusions

In summary, Guo *et al.*^17^ developed calixarene·LCG reporter pairs for selective neurotransmitter detection *via* an enzyme-coupled assay, enabling real-time enzyme monitoring and inhibitor screening. This work exemplified the evolution of supramolecular sensing from simple molecular recognition to multifunctional integration. While representing a significant advance, the authors also noted that it was currently limited to *in vitro* applications, with significant constraints *in vivo*, and issues such as salt interference and long-term stability under physiological conditions still needed to be resolved.

Future advancements in this field will require multidisciplinary collaboration to systematically address existing challenges. The integration of innovative supramolecular materials design with advanced characterization techniques will enhance the robustness and reliability of supramolecular sensing platforms under complex biological conditions. Furthermore, the development of novel macrocycles and advanced optical probes, such as the creation of advanced reporter pairs, can lead to theranostic supramolecular materials.^10,23^ Particularly, enzyme-regulated supramolecular chirality sensing and chiral detection will offer new dimensions of selectivity and enable the discrimination of enantiomeric biomolecules. Beyond sensing applications, these integrated materials would enable simultaneous imaging of pathological biomarkers and targeted therapy. These developments would ultimately advance detection capabilities from *in vitro* screening to real-time *in vivo* monitoring, potentially enabling dynamic tracking of metabolic processes in living systems.

## Author contributions

S. Q. S. and Y. L. wrote the manuscript.

## Conflicts of interest

There are no conflicts to declare.

## Data Availability

There is no additional data associated with this article.
